# Identification and manipulation of tumor associated macrophages in human cancers

**DOI:** 10.1186/1479-5876-9-216

**Published:** 2011-12-16

**Authors:** Moniek Heusinkveld, Sjoerd H van der Burg

**Affiliations:** 1 Dept. of Clinical Oncology, Leiden University Medical Center, Leiden, The Netherlands

**Keywords:** Tumor infiltrating macrophages, M1, M2, T cells, immunotherapy

## Abstract

Evading immune destruction and tumor promoting inflammation are important hallmarks in the development of cancer. Macrophages are present in most human tumors and are often associated with bad prognosis. Tumor associated macrophages come in many functional flavors ranging from what is known as classically activated macrophages (M1) associated with acute inflammation and T-cell immunity to immune suppressive macrophages (M2) associated with the promotion of tumor growth. The role of these functionally different myeloid cells is extensively studied in mice tumor models but dissimilarities in markers and receptors make the direct translation to human cancer difficult. This review focuses on recent reports discriminating the type of infiltrating macrophages in human tumors and the environmental cues present that steer their differentiation. Finally, immunotherapeutic approaches to interfere in this process are discussed.

## 1. Macrophages

Macrophages are a heterogeneous population of innate myeloid cells involved in health and disease. Macrophages originate from monocytic precursors in the blood and undergo specific differentiation depending on local cues in the tissue [1]. Roughly two distinct polarization states are recognized; the classically activated type 1 macrophages (M1) and the alternative activated type 2 macrophages (M2). In response to activating danger signals delivered by bacterial products or IFNγ, macrophages adapt to a M1 phenotype which is tailor-made to attract and activate cells of the adaptive immune system. Important features of M1 macrophages are the expression of iNOS, ROS and the production of NK and type 1 T-cell stimulating cytokine IL-12. M1 macrophages can phagocytose and kill target cells. M2 macrophages can develop in response to for instance IL-4 or IL-13[2,3], express abundant scavenger receptors and are associated with high production of IL-10, IL-1b, VEGF and matrix metalloproteinases (MMP). M2 macrophages play a role in parasite clearance and wound healing where they also polarize T cells to Th2 and dampen immune responses [4]. Furthermore macrophages are antigen presenting cells (APC) that express class I and class II HLA-molecules and co-stimulatory/inhibitory molecules to instruct T cells, albeit with lesser efficiency than dendritic cells. Notably, the terms M1 and M2 are an oversimplification of the macrophage types that can be detected. Macrophages display great plasticity and can adapt to a plethora of activation states ranging between the M1 or M2 phenotype depending on the mix of signals in their direct microenvironment. A number of excellent reviews on this topic were recently published [2,5-7]. In addition, fully polarized M1 and M2 macrophages can be redirected *in vitro *towards the opposite functional phenotype by treatment of the cells with cytokines [8]. For the remainder of this review we will refer to M1 and M2 macrophages under the notion that both M1 and M2 will reflect a whole range of functional states of macrophages.

## 2. Macrophages in human tumors; clinical impact & contrasts

For long macrophages are known to play a role in the development of tumors. This has been exemplified by their depletion in a number of mouse tumor models [9]. Carcinogenesis is characteristically associated with macrophage-mediated smoldering inflammation, often caused by pathogens [10], or as a result of autoimmunity and inflammatory conditions of uncertain origin [3,11]. In human studies on cancer, macrophages have predominantly been identified by immunohistochemistry applying antibodies against CD68 (Table 1) [12-52]. Most studies suggest that a high number of tumor associated macrophages is beneficial for tumor growth and, therefore, associated with disease progression and poor prognoses for the patient (reviewed in [53]). However, sometimes a high number of infiltrating macrophages correlates with better prognosis. For instance in human papilloma virus (HPV) induced cervical intraepithelial neoplasia macrophage infiltration was reported to correlate with disease progression [23]. Yet, in cervical cancers the number of stromal macrophages positively correlated with the intratumoral expression of IL-12p40, which itself was associated with a favorable overall survival of patients [54]. In lung cancer, higher numbers of IL-10+ CD68+ macrophages correlated with poor survival of patients with late stage disease [51]. However, another study showed that high macrophage numbers correlated with better survival. Importantly, in this study the polarization of the macrophages was determined and this revealed that improved survival was associated with a high M1/M2 ratio [37]. The type of macrophages studied thus may explain the apparent discrepancy between studies. Clearly, tumors can give rise to a heterogeneous population of tumor-infiltrating myeloid cells that differ at the molecular and functional level as a result of different instructions given by the local microenvironment [55-57]. The detection of macrophages on basis of CD68 does not allow identification of these distinct subsets and this deficit has been recognized in the field. Recent studies often show that antibodies to 2-3 different markers are combined (Table 1). Unfortunately, there are not many studies that apply the same set of markers to study the subsets of tumor-infiltrating macrophages. Furthermore, sometimes combinations of different antibodies were not used to discriminate between macrophage subsets but to differentiate between the expression of certain types of immune regulatory molecules by macrophages and tumor cells (*e.g*. PD-L1) [58,59]. It should be noted that the predominant detection of M2 macrophages reflects the late stage of tumor progression since the presence, type and role of macrophages in lower staged neoplasias is only marginally studied [23,60].

**Table 1:** Tumor associated macrophages in situ

Organ	Cancer type	n^1^	Method^2^	Markers	Macrophage infiltrate?	Link to prognosis^3^	Ref
Lymphnode	Hodgkins lymphoma	265	TMA IHC	CD68 CD163	Using a 5% of cells is positive cut-off; 80% is CD68+ cell positive and 64% is CD163+	No link	7

	Hodgkins lymphoma	130 and 166	IHC	CD68 MMP11	Using a 5% and 1% of cells is positive cut-off;72% is CD68+ cell positive and 41% positive for MMP-11	High CD68+ MF or high MMP-11 expression correlates with poor disease specific survival	40

	Hodgkins lymphoma	105	TMA IHC	CD68	42% has high MF infiltration (> 0,82% of total cells is positive for CD68)	High infiltration correlates with higher age and poor survival (also in the younger patients)	42

Colon	CRC	17	IHC	CCL2 CD68 IL-8	Mean MF number in tumor 8/mm3, in stroma 44/mm3 and necrotic area´s 44/mm3 and all tumors are high IL-8 postive	Tumor cell produced CCL2 correlates to MF count and advanced disease stage	8

	CRC	478	IHC	CD68	76% has high MF infiltration at tumor front	High MF count at tumorfront associates with better prognosis	16

	CRC	118	IHC	CD68 MMP-2 MMP-9	47% has high MF numbers and this correlates with expression of MMP-2 and MMP-9	Intratumoral TAM correlate with invasion, LN status and staging	21

	CRC	40	DIHC	CD68 S100 CD163	In all samples MF infiltration, more in stroma than in tumor beds. Mean is 12/15 per high power field.	Significant better survival for patient with high DC count and trend for high CD163 count.	29

	CRC	159	IHC	CD68 Clever-1/Stabilin-1 CD206 CD3+	98% has peritumoral macrophages in 10 tested tumors all MF are CD206/Clever-1 dubbelpositive	High peritumoral CD68 better prognosis; low intratumoral M1/M2 ratio more recurrent disease	52

	CRC metastasis in liver	15	IHC	CD68 CD163 CXCL-10 CXCR-4 CXCL-12 Her1 Her4 HB-EGF	CD68, CXCL-10, CD163 positive cells are found in al lesions	--	35

Intestinal tract	GIST	47 (19 metastatic)	FIHC	CD68 CD163 CD14 CD1a CD20 S100, HLA-DR CD3 CD8 FoxP3 CD33	High infiltrate in primary lesion tumors (2,7%) and metastatic lesions (4,9%) of CD163+ macrophages.	Presence of M2 is correlated with FoxP3 positive infiltrate	43

	gastric cancer	105	IHC ZG	hypoxia CD68 CD34 VEGF MMP2 +9	More macrophages in hypoxic area's	Hypoxia and more macrophages correlates with shorter survival	34

Lung	NSCLC	100	DIHC	CD68 HLA-DR CD163	Of CD68 positive MF 70% express CD163, 3% is positive for CD163 and HLA-DR	Patients with long survival have higher number of M1 type MF	28

	NSCLC	50	DIHC	CD68 IL-10	95% of IL-10 positive cells in advancing tumor margin is of MF origin	High IL-10 positive MF in advanced disease and therefore correlated to poor prognosis	46

	NSCLC	40	IHC	CD68 DR CD163 VEGF TNFa iNOS,	Less MF infiltrate in tumor islets of patient with shorter survival (all MF types)	More MF in tumor islets correlate with better prognosis	32

	NSCLC	20	IHC	CXCR-1,2,3 ,4,5 and CCL-1	CXCR-3 is positively correlated with MF number as determined in (ohri ERJ 2009)	Higher CXCR-2, CXCR-3 and CCL-1 expression in patients with extended survival	33

Mesothelioma	pleural mesothelioma	52 (+ 7 flowcytometry)	IHC	CD68 CD163 CD206 CD124	CD68+ MF comprised 27 % of tumor area, 7 tumors tested by flowcytometry; MF express high levels of M2 markers	High MF number in non-epithelial tumors is associated with bad prognosis	10

Breast	breast CA	110 + 106	IHC	CD68 anti-PCNA (proliferation)	Double positive proliferating macrophages are found in most tumors	High number of proliferating macrohpages associated with decreased survival	12

	breast CA	127	IHC	CD68 CD163 MAC387	Using a 25% surface area cut0off; 48% has high infiltration of CD163+ cells, MAC387 expression in 12%	High CD163 correlates with more distant disease recurrence	38

Breast and Colon	primairy metastatic lesions	49 breast and 12 colon metastasis	IHC	FCγRIIIa, FCγRII, CTSL1, CD163	Signature in primary lesion is the same as in corresponding metastatic lymphenode	--	44

Ovarian and Peritoneum	ovarium carcinoma		MS DIHC/FIHC	levels of eicosanoids (PGE2) and other related enzymes	High levels of eicosanoids in tumors, peritumoral CD163+ MF express COX and PGES	--	17

	epithelial ovarian carcinoma	40 (21 serous, 19 mucinous)	IHC	CD68 CD163 CD204 CSF-1	M2 cells present in stomal compartment of all patients, MF number and CSF-1 increase with disease progression	High M2 correlates with disease stage	22

	ovarium CA	60 +12	FIHC DIHC Flow	B7-H4 HAM56 CD3	70% of intratumoral MF express B7-H4	--	24

	ovarium CA	103	DIHC	B7H4 HAM56 (CD4 FoxP3)	Higher expression of B7-H4 on macrophages in advanced stage and correlate with the presence of regulatory T cells.	High B7-H4 expression on MF correlates with poor survival	25

Uterus	endometrioid carcinoma	64	IHC	CD163 CD31 HIF-1A	Only invasive tumors have high MF infiltration, more HIF1A and higher vessel density	In paired LN metastatic lesion same pattern for CD163 and CD31 is found	14

	endometrioid adeno CA	61	IHC	MMP-12 en CD68	Higher MF and MMP-12 expression in more advanced disease	Higher MMP-12 and MF in advanced disease	45

Cervix	CIN, HSIL, CxCa	86	IHC	CD68	Number of MF correlates with disease progression	High MF number predicts disease progression	18

Skin	stage 1/2 melanoma	227 blood 190 tumors	IHC ELISA	CD68 CD163 and soluble CD163	67% has dense infiltration with CD163 cells at invasive front and stroma	High CD163 in tumor front or stroma correlates with poor survival	20

	metastatic melanoma	6	FIHC	CD45 CD68 CD163 CD209 CXCL-12	60% of perivascular TAM expres CXCL-12	--	37

Prostate	first diagnostic screening;	92 of which 30% has prostate ca	IHC	CD68 CD204	Less CD204 positive cells is associated with development of prostate CA	--	31

	needle biopsies prostate	135	IHC	CD204	CD204 positive cells present in all samples	--	41

Eye	eye melanoma	43	FIH	CD68 CD163	Most MF are double positive, more macrophages in monosomy of chromosome 3	Less macrohpages is associated with better survival	9

Kidney	renal cell carcinoma	43	DIH	CD209 CD14 CD163	Most CD209+ cells were also CD14 and CD163+	--	15

Liver	hepatocellular carcinoma	63	IHC	CD68 B7-H1 (PD-L1)	High B7-H1 expression on tumorcells if high macrophage infiltration (48% of patients)	--	13

	intra-hepatic cholangiocarcinoma	39	IHC	CD68 CD163 CD34 FoxP3	50% of patients has high M2 infiltration and this correlates with vessel density and FoxP3+ numbers	High CD163+ correlates to poor disease free survival	19

Brain	glioma	79	DIHC	CD68 CD163 CD204 M-CSF	Higher macrophage infiltration and shift towards M2 type in advanced stage	More CD163+CD204+ macrophages and high M-CSF in advanced stages	23

Pancreas	pancreatic head cancer invasive ductal cancer	76	DIHC	CD68 CD163 CD204	42% shows high M2 infiltration perivasculair in invasive front	High M2 infiltration associated with LN metastasis	26

Soft tissues	leiomyosarcoma	76 gynecologic and 73 non- gynaecologic	TMA IHC	CD68 CD163	44% of patients has dense infiltrate with CD163+ cells	In non-gynecologic tumors, high CD163+ number correlate with poor prognosis	27

Peripheral Lymphoma	angioimmunoblastic T cell lymphoma	42	DIHC	CD68 CD163	Only invasive tumors have high MF infiltration, more HIF1A and higher vessel density	CD163/CD68 ratio correlates to overall survival	30

Thyroid	anaplastic carcinoma	27	IHC	NOX-2 P22-phox CD68 CD163	All tumors display a very dense network of TAM, 57% of tumor is TAM	--	11

	thyroid cancer	well/poor/anaplastic 33/37/20	IHC	CD68 CD163	27/54/95% has high CD 68 macrophage infiltrate thus more macrophage in advanced stage	--	36

Salivary gland	salivary gland	35	IHC	CD68 CD34 VEGFa	High infiltration with CD68 in 50% of patients	--	39

1. Number of patients analyzed

2. TMA=Tissue Micro Array, IHC= immunohistochemistry, DIHC = double staining for immunohistochemistry, FIHC=fluorescent immunohistochemisty staining, ZG= zymography MS=mass-spectometry, Flow=Flowcytometry

3. Macrophage infiltrate was correlated to survival, -- = not conducted

Alternatively, the number of tumor associated macrophages may form an epiphenomenon reflecting an inflamed tumor micro-environment in which many types of immune cells can be found [48,61]. This may comprise strong infiltration by regulatory T cells that exert a negative impact on clinical outcome. On the other hand it may also include the infiltration of tumors by T cells with cytotoxic function and a type 1 cytokine profile [62,63]. Moreover, macrophages do not only contribute to the local inflammation but also impair the activation of an effective anti-tumor T-cell response in tumor draining lymph nodes by inducing apoptosis [64].

## 3. Identification of distinct human macrophage subsets

Macrophages can be identified by the expression of transcriptional factors, cell surface markers, the production of cytokines and their function *in vitro*. In humans, the detection of tissue associated monocytes and macrophages *in situ*, is predominantly based on the use of antibodies to the glycoprotein CD68. However, antibodies to the LPS-co receptor CD14 or to HLA class II, represented by HLA-DR, are also used. Potentially, antibodies to the Fcγ-receptors can also be used, as these receptors - used to bind antibody-bound particles - are highly expressed by macrophages. So far, only antibodies to FcγRIII (CD16) have been used for the flow cytometric analysis of macrophages *in vitro *[65]. Newly added to the list of antibodies are those that target macrophage subset-specific markers. M2 macrophages express high levels of CD163, a hemoglobin-scavenger receptor, and can be used to discriminate between M1 and M2 macrophages (Table 1 and [66,67]). Tolerogenic macrophages and DC display higher levels of the mannose receptor (CD206) which is another scavenger [5]. In addition, the macrophage scavenging receptor 1 (CD204) is used as a specific M2 macrophage marker (Table 1). Thus far no unique markers for M1 macrophages are described. A list of markers which are currently applied to detect and distinguish between M1 and M2 macrophages is shown in table 2[5,6,29,53,67-77]. Matters, however, become more complicated when these macrophages become activated either via their pathogen-associated molecular pattern (PAMP)-receptors or upon cognate interaction with other immune cells. We and others have observed that activation results in loss of expression of the typical macrophage (*e.g*. CD14) and M2 markers (*e.g*. CD163, CD16 [65,66]). This implies that the use of a combination of different antibodies against cell surface markers to detect distinct macrophage subsets polarization will always be imperfect, albeit able to deliver much more information as in previous studies. To overcome this problem the expression of molecules that are directly related to macrophage function can be studied. Matrix metalloproteinases (MMPs) are proteolytic enzymes that degrade extracellular matrix and the basic membrane and are involved in tissue remodeling, tumor invasion and neovascularization of tumors. For instance, MMP 1, 2 and 9 are abundantly produced by M2 macrophages. The expression of MMPs in combination with CD68 or CD163 has been used to visualize M2 macrophages in tumors (Table 1). The functional activity of M1 macrophages is characterized by the production of reactive oxygen species (ROS) and nitric oxide (NO). However, due to their short life-span and therefore enzymatic activity these molecules are hard to detect. Instead, the detection of with this function associated enzymes like the inducible nitric oxide synthase (iNOS) is used in some of the studies (Table 1).

**Table 2:** Markers used to identify human tumor associated macrophages

Molecule	Function	*in situ*^1^	*in vitro*^2^		Expression^3^			
				**monocytes**	**M1**	**M2**	**other**	**ref**
				
CD68	Glycoprotein for adherence	IHC		x	x	x		53
CD14	LPS co-receptor	IHC	Flow	x	x	x		67.73
CD163	Scavenger receptor hemoglobulin	IHC	Flow	+/-	+/-	xx		5,63,75,76
CD206	Mannose receptor		Flow		x	xx		6
MMP-2	Matrix metalloproteinase	IHC	Digestion			x	Tumor cells	70
MMP-9	Matrix metalloproteinase	IHC	Digestion			x	Tumor cells	70
HLA-DR	Antigen presentation molecule	IHC	Flow	x	x	x	Immune cells	
CD204	Macrophage scavenger receptor 1	IHC			x	x	Tumor cells	71
B7H4	Inhibiting costimulatory molecule	IHC	Flow			x	Tumor cells	29
CD11b	Mac-1		Flow	x	x	x		73.76
FRb	Folate receptor beta		Flow			x		74
STAT-3	Transcription factor	IHC	Flow			x	Tumor tissue	77
iNOS	Nitric Oxide Synthase	IHC			x			72
IL-12p70	Interleukin	IHC	ELISA	x	xx			67.69
IL-10	Interleukin	IHC	ELISA	x	x	xx	Tumorcells	67

1. IHC = Immunohistochemical staining

2. Flow = immunofluorescent flowcytometry, ELISA = Enzyme-Linked Immunosorbent Assay, Digestion = collagen digestion assay

3. × = present on cell subset, XX = highly expressed or produced

In contrast to studies on macrophages *in situ *it is easier to assess macrophage function *in vitro*. All macrophages can phagocyte particles and apoptotic cells. Upon activation M1 macrophages will respond with an oxidative burst by the production of ROS and NO via iNOS as well as by the release of interleukin-12, known for its type 1 T-cell polarizing capacity. The use of NO to identify human M1 macrophages is somewhat debated as it was not detected in human macrophage cultures but abundantly found in tissues as detected by IHC (reviewed [72]). Therefore, *in vitro *studies on human macrophages focus mostly on the production of IL-12 and IL-10 to discriminate between M1 and M2 subsets [66,67,78,79].

New markers that may assist to discriminate M1 from M2 macrophages are typical transcription factors expressed by these cells [80]. M1 cells highly express IRF-5, a transcription factor that is involved in stimulating the production of type 1 interferon while repressing the production of IL-10. Forced expression of IRF-5 in M2 macrophages switches them to M1 macrophages. Genetic polymorphisms that induce overexpression of IRF-5 mRNA in human auto-immune pathologies like SLE and multiple sclerosis illustrate the link between high IRF-5 expression and type 1 inflammation in human beings (reviewed in [81,82]). Secondly, the signal transducer and activator of transcription (STAT) protein family is associated with the function of immune cells. Cytokine induced phosphorylation and nuclear expression of STAT1 is associated with a type 1 pro-inflammatory phenotype whereas STAT3 resembles an anti-inflammatory phenotype. STAT3 expression is often found in tumors and tumor-infiltrating macrophages and is induced by IL-6 and IL-10 [77].

Last but not least, macrophages produce chemokines to attract other immune cells and the type of these chemokines may offer help to distinguish between the macrophage subsets. For example, activated M1 macrophages produce CXCL-10 (IP-10) in order to attract CD8 and Th1 CD4+ T cells. M2 macrophages attract immune cells by the production of CCL-22 and CCL18. Differentiation between human M1 and M2 macrophages on the basis of their chemokine production has so far only been evaluated *in vitro *[18,79]. *In vivo*, most studies focused on the total tumor-derived chemokinome and, therefore, the exact role of macrophage-produced chemokines to attract immune cells is not clear yet.

It has to be said that there are a number of reports suggesting that also tumor cells and monocytes might express CD163, the detection of which depends on the antibody that is used [43,68]. Furthermore, MMPs are reported to be produced by tumors as well [50]. MMPs are thought to contribute to tumor progression, however, there is accumulating evidence that MMPs may also confer tumor protection [70]. In our opinion, careful assessment of macrophages in the tumor requires preferably the use of 3 markers to identify macrophages and their functional profile.

## 4. Induction of tumor associated macrophages

Tumors require nutrients, oxygen and the ability to discharge metabolic waste and carbon dioxide. These needs are addressed by tumor associated neovascularization [11]. Macrophages are the perfect help in this process, especially the wound-healing subset, since they are equipped to remodel tissue and produce VEGF. How tumors attract these myeloid cells has been excellently reviewed before [83].

CCL-2 produced by tumor cells and tumor associated cells attracts and shapes myeloid cells and interferes with osteoclasts in bone- metastatic disease [84,85]. Blocking CCL-2 reduced the number of tumor-infiltrating macrophages in pre-clinical animal studies. Secondly, the transcription factor Hypoxia Inducible Factor-1 (HIF-1), highly expressed under hypoxic conditions, regulates the attraction of monocytes and macrophages into the tumor amongst others by induction of Colony Stimulating Factor-1 (CSF-1/M-CSF) [83].

Many studies have been conducted to determine which factors induce macrophage differentiation upon arrival in the tumor. The levels of several cytokines were measured in tumor fluids, in ascites or in blood. Furthermore, the gene expression profiles of tumor cells have been analyzed. Interleukin-6 (IL-6) was often reported to be present in tumor fluids and was a produced by many tumor derived cell lines. IL-6 is a cytokine involved in chronic inflammatory diseases such as rheumatoid arthritis [86]. A number of studies on the *in vitro *differentiation of M2 macrophages - when monocytes or immature DC are exposed to tumor cell supernatant derived from lung, ovarian or cervical cancer cell lines - showed that this differentiation depended on IL-6. In nearly all cases IL-6 was shown to act in synergy with other factors [66,78,79,84,87]. Binding of IL-6 to its receptor results in activation of STAT3 in immune cells and consequently in the suppression of immune mediators that activate the immune response [77].

CSF-1 (M-CSF) mediates, besides being a chemo attractant, the differentiation of monocytes into CD14+CD163+CD206high M2 macrophages *in vitro *[67]. A similar dual role has been suggested for VEGF which acts as an angiogenic factor when secreted by wound healing macrophages but which can also impair APC differentiation when secreted by tumor cells [88].

The inflammation-inducible cyclo-oxygenase-2 (COX-2)-derived prostaglandin E2 (PGE2) is overexpressed by several human tumor-types [89]. The role of COX and PGE2 in oncogenesis has been well investigated in colorectal cancer. Blocking this pathway prevented oncogenesis in the low-intestines [90]. *In vitro *the addition of PGE2 to IL-4/GM-CSF stimulated monocytes skews their differentiation towards M2 macrophages [66,78,91,92]. Notably, overexpression of COX-2 in human lung cancer cells resulted in the production of IL-6 and the subsequent phosphorylation of STAT3. This rendered the tumor cells more resistant to apoptosis as well as increased their VEGF production [93].

Human tumors express and release high levels of Heat Shock Protein 27 (HSP27) (reviewed by [94]). The addition of soluble HSP27 to monocytes *in vitro *directed their differentiation to macrophages with an immune tolerizing and proangiogenic phenotype [95].

**Figure 1:**
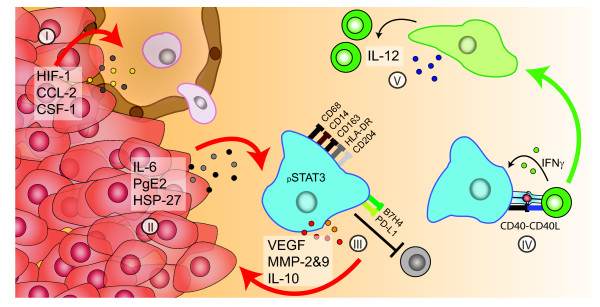
**Macrophages and T cells in the tumor micro milieu**. Tumors actively attract myeloid cells by the secretion of chemokines which is amongst others driven by hypoxia (I). Once myeloid cells have arrived in the tumor micro milieu several tumor cell produced factors drive the differentiation into a range of different types of macrophages, including M2 macrophages as a consequence of high STAT3 phosphorylation (II). M2 macrophages produce tumor promoting factors, express inhibitory molecules and upon activation produce anti-inflammatory cytokines and metalloproteinase that hamper T-cell function and promote tumor growth (III). Activation of M2 macrophages in the presence of IFNγ - for instance following cognate interaction CD4+ type 1 helper T cells - switch M2 macrophages to pro-inflammatory activated M1 macrophages (IV). M1 macrophages alter the immune suppressed micro milieu by producing IL-12 helping anti-tumor T cells as well as directly attack tumor cells (V).

Thus, inflammatory mediators attract myeloid cells to the tumor and direct their differentiation towards tumor promoting macrophages (Figure 1). The macrophage in the tumor tissue is subject to local levels of many factors that lead to a great variety in myeloid cell subsets even within a single tumor. Interestingly, most described factors are also produced by macrophages suggesting a feed forward loop.

## 5. Macrophages and adaptive immune cells in cancer

The plasticity of macrophages has been shown *in vitro *but whether M2 macrophages can revert to M1 and *vice verse *within the tumor environment is an emerging question. *In vitro*, exposure of murine macrophages to a type 1 cytokine environment skews them towards M1 macrophages. In contrast, the macrophages adapted a M2 phenotype upon exposure to IL-10 [8,96]. Adaptation to a particular phenotype was reversible by subsequent changes of the cytokine milieu [96]. Also human macrophages show great plasticity with respect to macrophage phenotype following stimulation with anti- or pro-inflammatory stimuli [97]. IFNγ, which was originally called macrophage activating factor, plays a major role in the differentiation towards M1 macrophages. It's produced by both innate and adaptive immune effector cells, including CD8 T cells and several subsets of CD4 T cells, NK cells as well as γδ T cells. IFNγ induces STAT1 and IRF-5 signaling-mediated type 1 cytokine production. Dulluc *et al*. comprehensively showed that IFNγ can prevent and reverse the M2-skewing effect of ascites from patients with ovarian cancer. Furthermore, pre-treatment of M2 macrophages with IFNγ results in a macrophage type that produces more IL-12 and less IL-10 upon activation with several innate stimuli [79].

Cognate interaction with CD4+ helper T cells constitutes a powerful activation signal for antigen presenting cells. The signal is delivered via CD40-CD40L interaction following T-cell receptor mediated MHC class II-presented antigen recognition. CD40L and IFNγ can activate in synergy but do not have to come from the same CD4 T cell. Exposure of APC to IFNγ-producing CD8+ T cells and CD40-L+ CD4 T cells allows APC to produce IL-12 and to become strong promoters of Th1 responses [98,99]. We showed recently that tumor skewed IL10-producing M2 macrophages can fully revert to IL12-producing M1 macrophages following interaction with CD4+ Th1 cells or with CD40-L-expressing cells in the presence of IFNγ [66]. Notably, in this *in vitro *study CD40-CD40L interaction resulted only in a strong activation signal for macrophages that was required but not sufficient to change the macrophage subset. Interestingly, we have observed that a minor population of tumor-infiltrating human CD8+ T cells expresses CD40-L upon activation [100]. This poses the question whether certain populations of intratumoral CD8 T cells can mediate the same effect on macrophages as CD4+ Th1 cells. The important role of T cells in instructing APC is further supported by studies showing that freshly isolated and activated blood-derived regulatory T cells skewed monocytes to M2 macrophages, which were able to produce high levels of IL-10 when stimulated with LPS [101]. The regulatory T cells, defined as CD4+CD25high cells, exerted their effect via the secretion of IL-13, IL-4 and most importantly IL-10 that was produced upon T-cell activation [101].

Mouse tumor-models support the significance of cognate interactions between CD4 T cells and macrophages. In an aggressive breast tumor-mouse model IL-4-producing CD4+ T lymphocytes indirectly promoted invasion and subsequent metastasis of mammary adenocarcinomas by regulating the phenotype and effector function of tumor-associated macrophages [102]. In another study, myeloma tumors were infiltrated by macrophages within 3 days, followed by tumor-specific CD4+ Th1 cells at day 6 after which the macrophages started to kill tumor cells [103]. This indicates that during early tumor development a Th1-macrophage response controls tumor outgrowth. Thus the type of T cell that interacts with tumor associated APC influences the polarization and function of the APC. Depending on the type of T cell, APC can swap their function allowing them to suppress or to stimulate immunity and *vice verse*.

Studies that evaluate the type of macrophages in relation to the number and type of T cells in patients are scarce. In ovarian cancer high numbers of B7H4 expressing immunosuppressive macrophages correlated with increased numbers of FoxP3+ T cells [30]. Accordingly, in gastrointestinal stromal tumors the number of CD163+M2 macrophages correlated with CD3+FoxP3+ regulatory cells [48]. A similar relation was reported for hepato-cholangiocarcinomas [24]. In contrast, in Hodgkin lymphoma an inverse correlation with CD68+ macrophages and FoxP3+ T cell numbers was reported. Here macrophages positively correlated with higher numbers of granzymeB and PD-1 expressing T cells instead. Alas, in this last study the type of infiltrating macrophages was not evaluated but the number of macrophages correlated to poor prognosis suggesting a M2 type of infiltration [47]. Finally, tumor-infiltrating macrophages were reported to attract IL-17+ CD4 T cells in liver carcinoma, thereby sustaining inflammation and supporting tumor promotion [104]. A recent study showed that a high number of macrophages and CD4+ T cells was correlated to a bad prognosis in breast carcinoma whereas CD8+ T cell dominance related to good prognosis [105]. Based on the MMTV-PyMT breast tumor mouse model, in which these authors also found a relation between macrophages, CD4 T cells and poor outcome, the CD4 T cells are likely to be Th2 cells [102]. Although these studies show that the number of infiltrating macrophages is related to the number of (regulatory) T cells it is not clear if these cells interact and what the outcome of interaction is with respect to macrophage function. For that the functional properties of all infiltrating T-cell subsets should be evaluated.

## 6 Repolarization of macrophages - a way to treat cancer?

The strong infiltration of human tumors by activated CD8+ cytotoxic lymphocytes (CTL) and CD4+ helper T (Th) cells but not immune suppressive cells such as regulatory T cells, M2 macrophages and myeloid derived suppressor cells is a hallmark for improved survival after therapy (reviewed by [106]). The treatment of cancer may take advantage of therapies that interfere with M2 macrophages, if combined with standard or immunotherapeutic regimens. Therapeutic modalities may attack at several levels; the attraction, the differentiation or the activation of macrophages.

One therapeutic option is to interfere at the level of macrophage attraction and differentiation by abrogation of the PGE2, IL-6 and STAT3 activation loop. This may directly affect tumor growth and limit the induction of tolerogenic macrophages as well. Several trials were conducted in which PGE2 production was inhibited by blocking of the COX-2 enzyme, often in combination with standard cytotoxic therapy [90,107]. Since all studies were initiated to block the paracrine loop on tumor cells not much is documented about the immunological effects let alone macrophage differentiation. One report mentions high immune cell infiltration, including macrophages upon 3 days of treatment with the COX-inhibitor [108].

A second strategy to interfere with immune cells in the tumor micro milieu is the blockade of cytokines secreted by the tumor or immune cells (*e.g*. M-CSF or IL-6). Recently, a phase II trial in patients with ovarian carcinoma was reported were the anti-IL-6 antibody siltuximab was tested [87]. The goal in this study was to deprive the tumor from growth factors and to prevent the differentiation of M2 macrophages. It might prove difficult to reach high enough antibody titers to block cytokine levels as macrophages tend to accumulate in poorly vascularized tumor areas [83]. Blocking of the cytokine receptors is not advised as membrane bound antibodies may activate type 2 macrophages via cross linking of the FcγReceptors and thereby activate tumor promoting cascades. This was recently demonstrated *in vitro *for the humanized Fc region optimized antibody cetuximab that binds the EGF-Receptor at the tumor cell surface and which caused activated M2 macrophages to produce IL-10 following the cross-linking of Fc-receptors [65].

All above mentioned strategies aim to inhibit the induction of M2 macrophages but do not reprogram M2 macrophages once they exist. *In vitro *macrophages can adapt to another phenotype upon a strong polarizing stimulus as discussed before [97]. Therefore, deliverance of a local APC-activating stimulus may be an attractive option. Macrophages highly express PAMP-receptors and several agonists for TLR and NOD-receptors are currently under clinical investigation for the treatment of cancer. For instance, easy accessible (pre-)malignancies have already been treated with TLR7 agonists [109]. We showed that activation of M2 macrophages *in vitro *by a number of different TLR agonist's results in the loss of expression of typical M2 markers but did not alter their typical M2 function [66]. Following this principle through made us realize that it also bears impact on other studies in this field. The group of Zitvogel extensively studied the effects of chemotherapy and showed that HMGB-1 released from dying tumor cells acted as a TLR4 agonist to activate APC [110]. The specific immune modulating properties of proteins released from dying tumor cells (e.g. HMGB-1) on the activation of M2 macrophages via PAMP-receptors is not yet studied.

Reprogramming of M2 to M1 type macrophages not only requires receptor-mediated activation signals but the presence of polarizing cytokines (*e.g*. IFNγ) as well. TLR are also expressed by T cells and stimulation modulates T-cell responses upon TCR triggering [111]. At the T-cell population level this may result in the enhanced production of IFNγ by T cells but also the downregulation of regulatory T-cell function [111]. One can envisage that if TLR-agonists not only activate macrophages but also T cells to produce IFNγ this will result in reprogramming of the macrophages. On the other hand, our own studies on the TLR expression by patient derived tumor-antigen-specific CD4+ T-cell clones, including helper T cells and regulatory T-cell clones, revealed that human T cells express a number of different TLRs at the mRNA level and that the TLR expression pattern differed per CD4+ T-cell clone independent of their function. When these CD4+ T-cell clones were tested in various activation (proliferation, cytokine production) and suppression assays no consistent gain (proliferation/IFNγ production) or loss in function was observed due to the presence of TLR-agonists either with or without TCR triggering. This suggests that TLR may not play a major role when CD4+ T cells are fully polarized already (unpublished observations). It is, therefore, questionable whether the application TLR agonists may result in repolarization of macrophages.

A recent study revealed that activation of macrophages by the infusion of antibodies against CD40 may induce macrophage-mediated tumor regression in 30% of cases in both a mouse model for pancreatic cancer and in patients with pancreatic cancer [112]. CD40 therapy resulted in the stimulation of secondary lymph node resident macrophages to migrate into the tumor tissue. Whereas, tumor-infiltrating in non-treated animals predominantly produced IL-10, the tumor-infiltrating macrophages of antibody treated mice produced IL-12 [112]. Notably, macrophages already present in the tumor were not stimulated by this treatment, possibly due to a low tissue penetration of the antibody [112]. *In vitro *CD40-activation with IFNγ was required to effectively reprogram tumor-induced M2-like macrophages into activated IL-12 producing M1 cells (Figure 1). As CD40 is not only expressed by macrophages but also by monocytes, endothelial cells, epithelial cells, and B cells [113], it is likely that the activation of several cell types throughout the body resulted in the deliverance of the necessary pro-inflammatory cytokines that allowed the circulating CD40-activated macrophages to adapt to a M1 phenotype.

In order to reprogram macrophages directly in the tumor-microenvironment, it is essential that CD4+ Th1 cells are locally present. The capacity of tumor-specific Th1 cells to directly alter the tumor microenvironment has also been recognized in studies on tissue-infiltrating CD8+ T cells in mice models. Th1 cells were essential for successful recruitment, local expansion and full effector function of large numbers of CTL by modulation of the local environment [114,115], and this may have included the repolarization of macrophages. In order to obtain sufficient numbers of tumor-specific CD4+ Th1 cells one may make use of adoptive T-cell transfer protocols [116,117] or apply strong vaccines [118,119].

## Conclusion and prospective

Macrophages play an important role in tumors. Depending on the mode of activation, they may promote tumor growth and suppress local immunity or attack tumor cells and sustain tumor immunity. Immunotherapeutic strategies to combat cancer should incorporate approaches focused on the attraction and polarization of M1 macrophages as well as on the reprogramming of M2 macrophages to the M1 subset. This requires a well-based understanding of the different subsets of macrophages in human tumors as well as their interaction with other members of the immune system, including T helper cells. To this end, the type and numbers of macrophages in the tumor have to be more carefully analyzed by the simultaneous use of several markers that allow discriminating the different macrophages subsets in one run. New and better subset-specific markers need to be identified. In addition, it will require in-depth studies on the interaction of local T cells and macrophages, a subject which currently is only marginally studied in human cancers.

## Abbreviations

M1: type 1 pro- inflammatory macrophage; M2: type 2 macrophage; DC: Dendritic Cell; CTL,Cytotoxic CD8+ T cell; Th: CD4+ helper T cell; ROS: reactive oxygen species (ROS); NO: Nitric Oxide; iNOS: inducible Nitric Oxide Synthase; HMGB-1,High Mobility Group protein B1; HSP: Heat Shock Protein; PAMP: Pathogen Associated Molecular Pattern; TLR: Toll Like Receptor.

## Competing interests

The authors declare that they have no competing interests.

## Authors' contributions

MH and SHvdB contributed equally to this manuscript. All authors read and approved the final manuscript.
